# Measurement of Quality of Life II. From the Philosophy of Life to Science

**DOI:** 10.1100/tsw.2003.76

**Published:** 2003-10-13

**Authors:** Soren Ventegodt, Joav Merrick, Niels Jorgen Andersen

**Affiliations:** The Quality of Life Research Center, Copenhagen K, Denmark

**Keywords:** Quality of Life, QOL, SEQOL, QOL5, QOL1, measurement, rating scale, combination scale, human development, holistic medicine, public health, Denmark

## Abstract

We believe it should be possible to make operational the philosophical ideas of the good life in order to make it the object of scientific research. The Quality of Life Research Center in Copenhagen, Denmark has therefore spent the last several years with these questions and tried to find practical and evidence-based scientific solutions.This paper describes the theoretical road taken in moving from the abstract philosophy of life to the actual questionnaire. It presents an important aspect of our work with the quality-of-life (QOL) concept though the last decade. We have developed the quality-of-life philosophy; the SEQOL, QOL5, and QOL1 questionnaires; the quality-of-life theory; and the quality-of-life research methodology. We carried out quality-of-life population surveys and developed techniques for improving quality of life with the chronically sick patient. This paper presents the struggle to create a rating scale for the generic measurement of the global quality of life, based on quality-of-life theory, derived from quality-of-life philosophy. The developed rating scale is a ratio scale combining a Likert scale, a visual analogue scale, and a numerical scale, to a reduced combination scale. This allows for the extraction of as much information from the respondents as possible without exhausting them unduly or demanding more than can be reasonably expected.

## INTRODUCTION

We believe that the philosophical ideas of life can become the object of scientific research. This is not an easy task, but as we have chosen the quantitative questionnaire as a tool, our task is now to spell out this philosophy and theory of life so that the abstract notions have the clarity and accuracy that we must expect from scientific instruments.

This paper describes the theoretical road taken in moving from the abstract philosophy of life to the actual questionnaire. It presents an important aspect of our work with the quality-of-life (QOL) concept though the last decade. We have developed the quality-of-life philosophy[1,2]; the SEQOL, QOL5, and QOL1 questionnaires[3,4,5]; the quality-of-life theory[4,5,6]; and the quality-of-life research methodology[6,7]. We have carried out quality-of-life population surveys[8,9,10,11,12,13] and developed techniques for improving quality of life with the chronically sick patient[14,15]. A comprehensive presentation of our research approach can be found in our paper[16].

## PHILOSOPHY OF LIFE IS BECOMING A THEORY

Philosophies of life are typically expressed in abstract terms and even in metaphors that lend themselves to numerous interpretations. They therefore often have a broad appeal, enabling everyone to find some truth within them. However, the richness of interpretation and ambiguity of meaning characteristic of an abstract philosophy of life is a serious hindrance for its use within a scientific setting. Science demands that words be used unambiguously.

A theory systematizes the field with which our philosophy of life is concerned by dividing life into various domains with the sharpest possible borders. This makes the philosophy more factual, but at the expense of a loss of depth and richness. For this reason, some people might find it less credible. For example, a basic philosophy of life states that humanity has a depth that must be respected if a good life is to be realized. This sounds reasonable to most people: a theory that clarifies this philosophy will divide life into a number of domains, a number of needs that will have to be fulfilled: the need for food, sex, family life, self-realization, etc. This clarification is based partly on subjective and culturally conditioned estimates and is bound to lead to disagreement: "Why these exact needs, and not others?" In this connection, the purpose of delimiting such life domains is to formulate questions. The following paragraphs attempt to establish life domains and deduce the questions.

The formulation of questions for use in a questionnaire is largely a matter of subjective judgment, and since the description of a given life domain becomes more rigid when expressed through a concise question, the loss in semantic richness is so much more pronounced. We might believe that a question, logically deduced, is so self-evident in its unbelievable simplicity so as to be a lie. This is very much part and parcel of questionnaires, in the same way that other scientific fields require simplification in operational processes.

What is the underlying aim of a question? As the concern here is the quality of life, a theory of the quality of life will indicate a number of areas in our lives that the overall philosophy deems to be important to the quality of life. The status of these life domains is investigated by asking a representative number of people suitable questions: how well are your needs for food, sex, social contact, etc. being fulfilled? Every life domain is then allocated a certain rating by every respondent representing life at that moment. By adding together the ratings from each life domain we calculate the quality of life of that person, as defined by the quality-of-life theory[1,2,4,5,6].

If, for example, the respondent is questioned about all the needs that the theory stipulates as being essential to the quality of life, a total score from the answers will reflect the person's own assessment of the fulfillment of these needs, and hence, by virtue of the theory, their quality of life. If the questions are to be workable, they have to be presented in everyday language that allows the respondent to grasp the intended meaning without difficulty. We must have optimal communication between the interviewer and respondent. If the respondent has to guess what a question means, something is wrong.

## ESTABLISHING A RATING SCALE

In a scientific context, the series of questions in a questionnaire (including procedures for scoring and summing the answers) is an instrument by which we gain knowledge of the area concerning the theory in question. Each instrument determines to what extent a certain unit occurs — weight, size, satisfaction, etc. Within physiology, it might be relevant to use traditional instruments: for example, a weighing scale to establish whether a given person is malnourished, a chemical method for measuring blood glucose concentration, or a dynamometer test to measure strength in the leg muscle. Researchers normally resort to asking questions only when they want to know how the group of people involved assess their own situation or when the life domain in question cannot, or indeed should not, be measured or assessed in any other way.

Our goal is that our survey should provide quantitative answers. As with more tangible measuring tools, measurement using questions must be based on an appropriate, and predetermined, rating scale. A device that measures weight is fine if we wish to measure the weight and state of nutrition of that person, but what scale can measure the quality of life? First and foremost, we must ensure that the scale relates naturally to the subject. This means, among other things, that its dimension (what it measures: weight, length, satisfaction, etc.) is relevant to the subject.

Perceiving and evaluating the external world by means of a measuring device is not an artificial, technical, and scientific process far removed from humanity, but rather something we do in our everyday lives, for instance when we informally evaluate the tone in our voices, the pitch with which we speak, color, distance, pressure, heat, as well as such psychological factors as feeling good, pain, and joy. This means that a randomly chosen instrument cannot satisfactorily measure the quality of life. Some scales reflect more appropriately than others how a person assesses his or her sensory impressions and state of being.

Furthermore, our rating scale for the quality of life must be carefully calibrated using suitable interval sizes. This should allow for the extraction of as much information from the respondents as possible without exhausting them unduly or demanding more than can be reasonably expected. Suitable intervals will also make the score sufficiently sensitive to allow the final score to be determined with some precision.

When the rating scale is functioning well, it enables the interviewer and respondent to communicate effectively, thus generating a reliable score. This means that two subsequent measurements of the same subject produce the same result. Thus, if a respondent is questioned about satisfaction with life, the result should be the same in two consecutive surveys, even if they have forgotten the precise wording of the answer they gave to the original question. (This presupposes that the satisfaction with life of that person has not changed in the meantime.)

Part of the purpose of having a theory is to establish rating scales with suitable response categories for each of the life domains the theory designates as being important for the quality of life. As we are concerned with quality-of-life theories, the theory contains a certain standard of a good life because it postulates that, if certain life domains are assessed in a certain way by the respondent, life is good.

When the theory indicates that more or less objective areas are central to the quality of life, such as a vast number of friends or a high income, the indicator is usually self-evident: number of friends or income. If, however, we want the respondent to assess a subject qualitatively (How satisfied are you in your love relationship? Are you satisfied at work?), we need to consider carefully how to rate such findings.

If we wish to explore the quality of life of many people in order to make a comparison and deduce the quality of life of an entire population, the ideal scale to use would be a fraction scale[17,18]. A fraction scale spans from a natural bottom to a natural top, for instance 0 to 1 or 0% to 100%. The intervals can be interpreted as percentages of the maximum value of the scale. It is thus a ratio scale from both extremes. If a true fraction scale cannot be used, we must at least use an interval scale: a measuring device with equal distances between the intervals, thus enabling us to calculate averages, though not fractions.

A quality-of-life rating scale should have a neutral point (zero point) around which good and poor quality of life can be placed as positive and negative values, respectively.

## A NEW TYPE OF SCALE FOR RATING QUALITY OF LIFE

For the purpose of our surveys, we created a simple fraction scale with a zero point. In our earlier surveys we used a combination scale, combining three scales that were all well known in medical and social science literature, but they all have their own weaknesses: Likert scales, visual analogue scales, and numerical scales. We then reduced the combination scale formed by these three types of scales into a somewhat simpler model. This model should be particularly well suited for the questions that require a qualitative assessment (two thirds of the questions in our SEQOL questionnaire[3]).

*Likert scales* let the respondent choose between discrete, linguistically meaningful options, such as "very good", "good", "fair", and "poor". These options are not proportional to each other. There is no zero point around which the positive and the negative responses may rest. It is difficult to say whether there are natural intervals between the response options. They are certainly not of equal size. Therefore, we cannot deduce anything with precision from the score if we base it on a Likert scale. But we are close to everyday language, which is a prerequisite if the respondent is to understand the meaning of the question and hence supply us with the score we are seeking. Experience tells us that we get the best quality information when we use everyday language.

*Visual analogue scales* use horizontal lines, usually 10 cm long, representing a spectrum from, for example, "very good" to "very poor". Where appropriate, the respondents mark their evaluation of the subject on the scale. As this type of scale does not contain a limited number of possible responses, the respondents are given greater freedom of expression than a Likert scale can provide. However, since visual analogue scales do not have any intermediate points, such as "good" or "fairly good", the respondent cannot be sure whether the pole score "very good" actually means "quite good" or "exceptionally good". The inclusion of intermediate points would help the respondent in clarifying the meaning of the two poles.

*Numerical scales,* for example, the numbers from 1 to 10, let the respondent choose a number as an expression of his or her evaluation. The absence of verbalized categories makes the meaning of each number unclear. In other words, the respondent can make a fairly free choice, and that in itself is a great problem.

The advantage of visual analogue scales and numerical scales is their natural neutral point (in the middle) and their symmetry. It is an additional advantage that we can justify having equidistant numerical differences that correspond to a proportional change in the overall picture of the quality of life[6]. Visual analogue scales and numerical scales thus have the qualities of the interval scale.

Is it then possible to combine the scales so that the advantages are maintained and the less undesirable qualities are reduced? We experimented and created a scale we call a combination scale (see below in Table 1). It has the continuous line of visual analogue scales; the useful division of equidistant intermediary positions of numerical scales; and the designation of certain selected positions and categories of Likert scales. The 11 numbers and the 5 denoted categories have a natural neutral point, or middle value 5, around which the scale is also symmetrical.

**TABLE 1**
**The Combination Scale, Optimal to Extract Information from the Respondent Completing a Quality-of-Life Questionnaire**

**A Likert scale**. How are you feeling now? (check a box)

- ☐ Very good
- ☐ Good
- ☐ Fair
- ☐ Poor

**A Visual Analogue Scale.** How are you feeling now? (place an X on the line)

Very poor ________________________________________________ Very good

**A Numerical Scale** (a hidden visual analogue scale). How are you feeling now? (circle a number)

| Very poor | | | | | | | | | | Very good |
|---|---|---|---|---|---|---|---|---|---|---|
| 0 | 1 | 2 | 3 | 4 | 5 | 6 | 7 | 8 | 9 | 10 |

**A Combination Scale**. How are you feeling now? (circle a number)

| Worst possible | Quite poor | | | | Fair | | Quite good | | | Best possible |
|---|---|---|---|---|---|---|---|---|---|---|
| 0 | 1 | 2 | 3 | 4 | 5 | 6 | 7 | 8 | 9 | 10 |

The poor ratings are 1, 2, and 3; the intermediate ratings 4, 5, and 6; and the good ratings 7, 8, and 9. The extreme ratings 0 and 10 are rare.

In deciding your rating, you should first decide which of the three groups of ratings you prefer (or the extremes of 0 or 10). You can then choose the number within the group that suits you.

**The Reduced Combination Scale**. How are you feeling now? (circle a number)

| Visual interpretation | | The simplest numerical interpretation | Corresponds to the combination scale score | Interpretation of the combination scale (% of theoretical max.) |
|---|---|---|---|---|
| 1 Very good | } | +2 ±½ | 9 | 90% ± 10% |
| 2 Good | } | +1 ±½ | 7 | 70% ± 10% |
| 3 Neither good nor poor | } | 0 ±½ | 5 | 50% ± 10% |
| 4 Poor | } | -1 ±½ | 3 | 30% ± 10% |
| 5 Very poor | } | -2 ±½ | 1 | 10% ± 10% |

The zero point and the two final points, which constitute the top and bottom of the scale (0 and 100%, respectively, or 0 and 1), meant that we had obtained the qualities of the ratio scale at both extremes: not only can we state that a score of 0.4 is twice as high as 0.2 (twice as far from the final point of 0), but also that the distance from 0.4 to the maximum is twice as long as the distance from 0.7 to the maximum (being twice as far [0.6 vs. 0.3] from the final point of 1).

The scale makes the respondent divide the life domain to which the question relates into 11 options, which provides a rating in deciles of the theoretical span between minimum and maximum. This scale thus meets the methodological requirements for a rating scale[6,16,18].

We used this scale in a pilot survey of 200 residents in Denmark, randomly chosen from the Civil Registration System (CPR register). Only 50% of those 20–40 years old were able to reply precisely to such a complex scale even after receiving written instructions. We concluded that the scale had to be simplified, preferably without a loss of information other than what resulted from reducing the number of response categories. It was also crucial that, after the amendments, it would still be possible to interpret the responses against the original combination scale.

Table 1 shows the amended and final version of the scale, illustrated by the question from the questionnaire that assesses well being. The reduced combination scale has five equidistant categories from minimum to maximum, from which the respondent may choose: "very poor", "poor", "neither good nor poor", "good", and "very good".

These categories can be expressed as numerical values in different equivalent ways. If we focus on the symmetrical qualities of the scale, then its central neutral point, 0, will be defined as "neither good nor poor". The minimum ("very poor") and maximum ("very good") can then be represented numerically as –2 and +2, respectively, and "poor" and "good"" as –1 and +1, respectively.

If, however, the ratio qualities of the scale and its two extreme points, 0 and 100%, are to be emphasized, the five points should be scored as 10, 30, 50, 70, and 90%. These five categories then represent the quality of life (assessed according to a given quality-of-life rating scale) as a percentage of the theoretical maximum. Each of these five categories is presumed to have a factor of uncertainty on either side, corresponding to one tenth of the scale (0.5 or 10% in the two types of score, respectively). It follows that the minimum can be described by –2 or 10%, respectively, and not at the very tip (the zero point) of the scale. The reduced combination scale is so simple that everybody can use it, and it generates almost as much information as the original combination scale.

Is the simple, numerical interpretation shown above reasonable? Is "very good" (equal to 2) really twice as good as "good" (which has a numerical value of 1)? This would mean that "90%" is positioned twice as far from the zero point of "50%" as "70%" is. Isolated from the context of the scale, it is misleading. For instance, the Danish equivalent of "very good" in everyday speech tends to refer to something less than "good". However, when placed on a scale symmetrical around a natural neutral point and the adverb "very" as a modifier at both ends of the scale, the interpretations already mentioned are reasonable. The empirical ratio thus seems to correspond with the numerical interpretations of the responses, as linear regression is possible in most instances.

The expression "neither/nor" was chosen as the neutral point instead of other neutral alternatives. This is because "neither/nor" is a true neutral point as opposed to "fair" and because the responses do not cluster at the neutral point. This cluster may occur because life is so full of contradictions that other alternatives become too attractive. If this is the case, the responses lose their value.

Even if the response options chosen do not appear to be entirely satisfactory linguistically, as the discussion of "very good" and "good" shows, our studies showed that the respondent can easily understand the options when they see how the scale is constructed. This is part of our philosophy that the questionnaire must be meaningful to the respondent. The reduced combination scale meets our criterion to show quantitatively interpretable response options just as well as the original combination scale.

Let us admit that the reduced combination scale looks quite familiar. It has been used many times before, in exactly the same form as we present it in Table 1, as it is often intuitively chosen among many possible scales for its aesthetic appeal. What is new is the systematic construction of the scale by combining the scales most often used in psychometric studies, indicating that the reduced combination scale, attached to a question derived from a quality-of-life theory, makes the best possible quality-of-life rating scale. The QOL1, QOL5, and SEQOL questionnaires using this scale and principle have all shown to be valid and highly sensitive[3,4].

## THE RATING SCALE IN OPERATION

The appropriate rating scale is then applied to the relevant questions, which have to be constructed with suitable response options. These have to be worded in a natural, straightforward way so that the respondents will not be given so many response options that they become tired or confused. Nevertheless, there must be enough options to satisfy the desires of the average respondent, so that they will not feel the desire to place their reply in an intermediate category that does not exist.

The combination scale in this survey is used solely for questions assessing quality. These are all structured, as outlined above, with five response options: a neutral point, two extremes expressed with "very", and in between these "good" and "poor" (in questions regarding how one feels about something), or "satisfied" and "dissatisfied" (in questions regarding how satisfied one is with something), or "happy" and "unhappy", etc.

In five rows of questions, each spanning a narrower field than the quality of life (health problems, sexual problems, view of self, view of life, and values), the number of response options is reduced to three to make it easier to arrive at an answer. For four of these rows, a typical question might be "Do you accept yourself"? The reply is either a “yes” or a “no”, symmetrically placed around a central point: "don't know". The fifth row of questions pertaining to health problems (for instance, "Do you have difficulty falling asleep"?) gives the responses either "No", "Yes, a bit", and "Yes, a lot". Here there is no neutral point and no symmetry.

The responses for all five rows can be interpreted quantitatively on a percentage scale. In instances with three response options, the span from 0 to 100% is divided into equally large uncertainty zones, one on either side of each response option, which is 3 times 2 zones = 6. Hence: 100% divided by 6 = 16.67, which places the three response options at 16.67, 50.00, and 83.33%, respectively.

## HOW TO WEIGHT THE RESULTS

If more information is desired from a particular area of study, more than one question is required to generate thorough and varied responses. If one wishes to combine the responses to several questions into a rating of a particular domain of life, the weighting of each response in the overall rating must be considered carefully. This is expressed as a weight, a factor between 0 and 1, which is then multiplied by the quantitative score of each response. This number is then added to other responses within the life domain.

One of the eight rating scales of the Quality of Life Survey determines how well subjects feel during the three time phases into which a modern life is divided: work, domestic life, and leisure time (weekends and holidays). The total rating is a weighted combination of the three responses, and the weighting is one-third on all three. For example: "very good" ($0.33 \times 90\%$) + "poorly" ($0.33 \times 30\%$) + "good" ($0.33 \times 70\%$) = 63.33%, which is slightly below "good". This score then shows the total quality of life of this person according to the rating scale on job, family, and leisure time.

The weighting must follow the theory so that it is compatible with the overall philosophy of life of the theory. Weighting must not be random, nor must it be calculated solely statistically once the responses have been given, because then the results will be difficult to interpret.

A typical weighting problem when planning a quality-of-life questionnaire can arise when investigators have a specific area of interest as a result of the nature of their work. For instance, a nutritionist is interested in diet and may therefore have 30 questions that relate to diet. To achieve a comprehensive view, the nutritionist may add a further 10 questions on other aspects of life, such as mental health and social behavior. Without thinking about it, all questions are then summed and the total divided by 40. This means that every response has been weighted equally.

The effect of this is that the quality-of-life scale gives diet three times the weight of mental and social functioning.

Such a scale can be practical or impractical for the person who constructs or uses it. It raises the question of validity: whether the applied weighting correctly expresses the philosophy or understanding of human life underlying the survey as a whole. Even a nutritionist should be able to see that diet is not three times as important in a person’s life as mental and social functioning.

## CONSIDERATIONS OF LAYOUT AND PRESENTATION

A difficult part of developing a questionnaire is the aesthetic presentation: How does it feel in people's hands? Does the message get across? Do people understand right from the start what the purpose of the survey is and what we want from them? Is it clear from page one of the questionnaire the domains of life on which we wish them to report? Does poor layout influence the respondents and make them confused? Are the respondents influenced by inappropriate response sequences, poorly worded questions, or sensitive or taboo topics far too early in the questionnaire? Is there a natural development in the thoughts evoked in the respondent as he or she progresses with the responses? Do we force people to confront aspects of themselves that they wish to avoid, in ways that provoke defense, which again might lead to deceptive responses? Do we risk not getting any response at all?

Layout and visual and tactile impression also need to be considered. Is the concentration of the respondent ruined by inappropriate tables or by unusual, ugly, or demanding fonts? Are there uneven, wavy margins that promote lack of concentration, making it difficult to focus on titles and locate the questions? Is the paper right, the print nice, clean, and clear? Is the jacket pleasant to touch, the space for the responses easy to fill in? Is everything clear and detailed?

The layout is extremely important. It is often just as important a task as drawing up the questions for the questionnaire. We spent almost 3 years, and made 20 pilot versions, in preparing the questionnaire for the Quality of Life Survey. In the final phases we consulted external consultants with regard to choice of printing type, paper, presentation, and layout.

There are many more aesthetic and irrational factors: Is the language of the questionnaire sufficiently polite for elderly people and sufficiently informal for young people? Should one address people with the formal pronoun *De* (you in Danish has two versions) or the informal *du* when asking about their sex life? Does the choice of words match the subject and target group? Does the general philosophy of the survey generate sufficient confidence so that people dare respond to very personal questions about their view of self and sexuality? Is there accordance between the presentation of the survey and the image of the institution responsible for it? Is the envelope nice, with a suitable text? Is the logo of the survey appropriate? Is the cover letter written on the right type of paper and signed by the right people, thus giving it a seal of credibility? Is the time of year for the survey well chosen? Summer holidays and Christmas are not ideal. Does the survey coincide with a suitable mood and trend at the time so as to make it appealing, or might it be better to delay it until a more appropriate time? Does the survey coincide with a general election or the like?

If the mass media can be persuaded to cover the survey, the response rate might increase somewhat. But is it a good idea to bring forth a message that might cause a serious debate when such a public debate, and criticism, is bound to ruin the survey from the start?

The scope of a questionnaire-based survey is as wide as it is made out to be. The researcher's scientific integrity and sense of proportion and timing must determine the moment when the questionnaire is ready and can be given to the final group of respondents. The survey will never be better than the questionnaire sent off in the mail. One is always likely to regret later if one has been overly rash.

## ACKNOWLEDGMENTS

This study was supported by grants from The 1991 Pharmacy Foundation, as well as by supplementary grants from Goodwill-fonden, the JL-Foundation, E. Danielsen and Wife's Foundation, Emmerick Meyer's Trust, the Frimodt-Heineken Foundation, the Hede Nielsen Family Foundation, Petrus Andersens Fond, Wholesaler C.P. Frederiksens Study Trust, Else & Mogens Wedell-Wedellsborg's Foundation, and IMK Almene Fond. We gratefully acknowledge the critical scrutiny and expert linguistic assistance of Ib Ravn, Ph.D. The research was approved by the Copenhagen Scientific Ethical Committee under number (KF)V.100.2123/91.

## BIOSKETCHES

**Søren Ventegodt, MD,** is the Director of the Quality of Life Research Center in Copenhagen, Denmark. He is also responsible for a Research Clinic for Holistic Medicine in Copenhagen and is a popular speaker throughout Scandinavia. He has published numerous scientific or popular articles and a number of books on holistic medicine, quality of life, and quality of working life. His most important scientific contributions are the comprehensive SEQOL questionnaire, the very short QOL5 questionnaire, the integrated quality-of-life (IQOL) theory, the holistic process theory, the life mission theory, and the Danish Quality of Life Research Survey, 1991–94 in cooperation with the University Hospital of Copenhagen and the late pediatric professor Bengt Zachau-Christiansen. E-mail: ventegodt@livskvalitet.org. Website: www.livskvalitet.org/

**Joav Merrick, MD, DMSc,** is Professor of Child Health and Human Development affiliated with the Zusman Child Development Center and Division of Community Health at the Ben Gurion University, Beer-Sheva, Israel and presently the Medical Director of the Division for Mental Retardation, Ministry of Social Affairs, Jerusalem and the Director of the National Institute of Child Health and Human Development. He has numerous publications in the field of child and human development, rehabilitation, intellectual disability, disability, health, welfare, abuse, advocacy and prevention. Dr. Merrick received the Peter Sabroe Child Award for outstanding work on behalf of Danish Children in 1985 and the International LEGO-Prize ("The Children's Nobel Prize") for an extraordinary contribution towards improvement in child welfare and well being in 1987. E-mail: jmerrick@internet-zahav.net. Website: www.nichd-israel.com

**Niels Jørgen Andersen, MSc,** Professor, Department of Innovation and Economic Organization, Norwegian School of Management. This department conducts research and provides teaching in central topics related to innovation, business development, management of global companies, business history, and economic organization. Research activities within the Department are related to four core subjects within the discipline: business history, cooperative organizations, business development and entrepreneurship, and finally studies of industries with a special focus on the electricity industry. He is also the dynamic chairman of the nonprofit organization Stiftelsen Holistisk Medisin Scandinavia, which aims to support the scientific development, research, and documentation of complementary and holistic medicine in Scandinavia. E-mail: niels.j.andersen@bi.no. Website: www.bi.no/users/fgl93013/
